# Pathophysiological mechanisms and emerging therapeutic strategies for muscle wasting: an integrative review

**DOI:** 10.3389/fphys.2025.1674892

**Published:** 2025-10-27

**Authors:** Jiahao Huang, Tianshan Wen, Huijun Wei, Jiliang Kang, Junyue Lu, Taian Zhou, Youliang Wen, Haoyuan Huang

**Affiliations:** ^1^ School of Rehabilitation Medicine, Gannan Medical University, Ganzhou, China; ^2^ Department of Rehabilitation Medicine, Third Affiliated Hospital of Gannan Medical University, Ganzhou, China

**Keywords:** sarcopenia, cachexia, muscle atrophy, proteolysis, myostatin, regenerative medicine

## Abstract

Muscle wasting is a continuum of diseases that entail incremental skeletal muscle mass, as well as functionality, loss and is an important cause of morbidity, mortality, as well as quality of life reduction. This pathophysiology, diagnosis, as well as emerging treatment of significant muscle wasting illnesses, that is, sarcopenia, cachexia, disuse muscle atrophy, as well as neuromuscular illnesses, entail intricate interactions of defective protein synthetic processes, upregulated proteolysis, inflammatory cytokine activation, defective mitochondria, as well as hormone disturbances. Diagnostic methodologies have progressed from crude body dimension measures to sophisticated imaging modalities, as well as molecular biomarkers, but standardization remains contentious. Treatments entail targeted nutrition, as well as exercise regimes, as well as emerging drugs, as well as regenerative medicine therapies. Preclinical-to-clinical translation gaps, even striking, still exist despite promising advances. Some of these include diagnosis-based inequalities, patients' heterogeneity, limited therapeutic advantages, as well as implementation difficulties within healthcare. Future directions have emphasis on personalized medicine strategies that entail multi-omics signatures, combinatorial therapy of several targets, electronic health platforms for dynamic surveillance, as well as prevention modalities. Integrated healthcare platforms, multinational collaborative platforms, as well as regulatory reforms favoring muscle health across the life continuum, are needed for accomplishing the emerging challenge of controlling emerging muscle wasting burdens of the old, as well as those beset by chronic illnesses.

## 1 Introduction

Muscle wastage, or slowly decreasing skeletal muscle mass, power, and functioning, is one of the great clinical problems of enormous consequence for morbidity, mortality, and quality of life for various groups of patients (Cruz-Jentoft and Sayer, 2019). This disabling symptom is composed of a continuum of ailments: sarcopenia, cachexia, disuse atrophy, and neuromuscular disease-related muscle loss, each of these possessing distinguishable pathophysiology but molecular mechanisms also shared (Sayer et al., 2024). Muscle wastage is an ever-exponentially increasing health problem as an adjunct of an aging population as well as the ever-increasing incidence of chronic ailments, providing an imperative for extensive research of its pathogenesis as well as development of effective therapeutic leads. Sarcopenia, muscle strength and muscle mass loss that is detectable across ages, has also been codified as an International Classification of Diseases (ICD-10-CM) code, and hence is an exclusive diagnosis of geriatrics. Prevalence of sarcopenia is 10%–20% for people 60 or more years old, but does increase very quickly for higher ages and accompanying illnesses (Chen et al., 2020). Consensus statements have also made threshold values of diagnosis, such as muscle power, number or quality of the muscle, and physical performance, more streamlined clinically, thus better identifying and managing sarcopenia. Asian Working Group for Sarcopenia has even established these threshold values considering ethnic differences of body composition and functional measures, reiterating population-specific measures. In addition to ageing-related muscleatrophy, cachexia is a multi-metabolic condition of involuntaryweight loss and muscle catabolism that is recognized as an aftermath of chronic ailments such as cancer, heart failure, chronic kidney disease, and chronic obstructive pulmonary disease (Ebner et al., 2020). Unlike sarcopenia, cachexia is associated with systemic inflammatory response and metabolic dysregulation, which is not entirely corrigible by nutritional means alone, and raises special challenges for treatment. Multidimensional etiology of muscle catabolism also includes disuseatrophy as an aftermath of physical inactivity or immobility, and neuromuscular disorders due to progressive motor neuron or muscle fibre degeneration (Liu et al., 2024).

Pathophysiologic muscle wasting processes entail complex molecular networks of balance between protein synthetic and breakdown process (Wu et al., 2023). Key to these processes is anabolic dysregulation of the insulin-like growth factor-1 (IGF-1)/PI3K/Akt/mTOR signaling cascade, ubiquitinated by simultaneous activation of the ubiquitin-proteasome pathway and autophagy-lysosome machinery. Inflammatory cytokines, including tumor necrosis factor-alpha (TNF-α), interleukin-6 (IL-6), and interferon-gamma, orchestrate catabolic signaling, repressing anabolic responses, and thereby establish the metabolic conditions placing individuals at risk for muscle loss (Shen et al., 2024). Recent transcriptomic signatures have put forth immunocyte infiltration and inflammatory gene networks as being core to sarcopenia pathogenesis, establishing likely therapeutic foci. Myostatin, a member of the transforming growth factor-beta superfamily, has been identified as the primary adverse modulator of muscle volume, and heightened levels have been reported for several muscle-wasting disorders (Abati et al., 2022). Myostatin inhibition has brought enormous boost for its therapeutic use, but preclinical potency is frustratingly reluctant to transform into clinical outcomes. Additionally, mitochondrial impairment, oxidative injury, hormonal alterations, as well as neuromuscular connectivity defects, also play a critical role in the multi-pathophysiology of muscle wasting, for which multitargeted therapeutic strategies are required. Accurate diagnosis of wastedness of muscle is reached by the combination of functional tests, imaging modalities, and novel biomarkers (Li L. et al., 2024). While traditional methods use anthropometric measures and also traditional tests of function such as handgrip strength and walking speed, advances of modern technology of imaging have also heightened accuracy of muscle mass as well as quality assessment. Dual-energy X-ray absorptiometry, computed tomography, magnetic resonance imaging, and ultrasonography, offer adjunctive means of muscle quantity assessment and for discerning preclinical suggestions of disability such as intramuscular fat infiltiration (Chianca et al., 2022). Advances of novel circulating biomarkers, such as metabolomic signatures as well as microRNAs, have the promise of better early diagnosis as well as monitoring of therapy response (Hsu et al., 2024). Selected representative biomarkers associated with systemic inflammation, metabolic dysregulation, and muscle regeneration are summarized in Table 1.

**TABLE 1:** Selected circulating biomarkers associated with muscle wasting disorders.

Category	Examples	Pathophysiological relevance	Recent references
Classical inflammatory markers	C-reactive protein (CRP), Interleukin-6 (IL-6), TNF-α	Indicators of systemic inflammation driving proteolysis and anabolic resistance	Ebner et al. (2020), Kramerova et al. (2020)
Metabolic indicators	3-methylhistidine (urinary), creatinine, albumin	Reflect rates of muscle protein breakdown and whole-body catabolism	Hsu et al. (2024), Dudhat et al. (2025)
Lipidomic biomarkers	Ceramides, diacylglycerols, sphingolipid subclasses	Indicate lipotoxicity, insulin resistance, and inflammation in sarcopenia	Hsu et al. (2024), Kramerova et al. (2020)
Novel circulating miRNAs	miR-206, miR-1, miR-133a, miR-486	Sensitive regulators of muscle regeneration, proteolysis, and anabolic resistance	Kramerova et al. (2020), Fonseca et al. (2020)
Emerging composite ratios	IL-6/albumin ratio	Combines inflammation and nutrition markers, improving diagnostic specificity	Hsu et al. (2024), Fonseca et al. (2020)

The past several years have also seen remarkable progress in therapeutic measures for treating muscle wasting, from nutritional supplements and exercise-centered physical therapy to new drugs and regenerative therapies (Beaudry et al., 2022). Translating promising preclinical results into bedside applications, however, has been difficult for most measures, as numerous measures have exhibited limited functional enhancements despite muscle growth increments (Tsai, 2024). Difficulty associated with treating complex muscle wasting, along with the problem of patient heterogeneity and the necessity for individually tailored treatment regimens, emphasizes the continued necessity for research, as well as cross-disciplinary interactions. It is an integrated, broad-range literature analysis of what is out there about the pathophysiology, diagnosis, and new treatment of muscle wastage. Attempting to bridge perspectives between molecular biology, clinical, and translation literature, the analysis makes an attempt to draw out insights which can inform future directions of research and better management of patients suffering the debilitating condition.

## 2 Etiologies and classifications of muscle wasting

### 2.1 Sarcopenia

**FIGURE 1:**
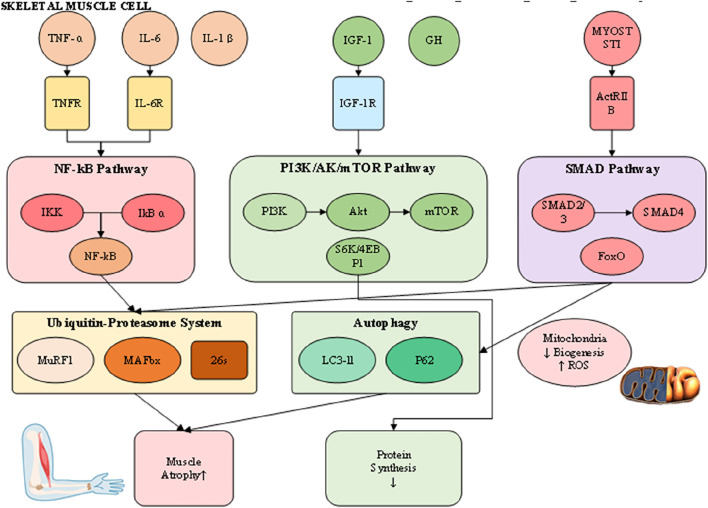
Classification of muscle wasting disorders.

Muscle wasting is an overarching term of numerous conditions of gradually progressive skeletal muscle mass and capacity loss that are caused by variable etiological origins ranging from physiological ageing to diverse general body illnesses (Sayer et al., 2024). It is extremely obligatory to recognize the classificaion as well as the cause of muscle wasting for the formation of etiology-specific treatment strategies as well as for maximising management measures clinically. Sarcopenia, cachexia, disuse atrophy, and neuromuscular disease-related atrophy comprise the four predominant categories of muscle wasting, each of which has unique pathophysiology but common typical molecular mechanisms (Figure 1). Sarcopenia, from the Greek term “sarx” (flesh) and “penia” (loss), is the age-related loss of skeletal muscle mass, strength, and physical functioning (Cruz-Jentoft and Sayer, 2019). Sarcopenia is one of the most prominent geriatric syndromes, and indeed, it has gained significant attention as an emerging major public health problem that affects 10%–20% of elderly individuals, and its occurrence escalates sharply with growing old (Chen et al., 2020). Sarcopenia pathogenesis is composed of several intertwined processes including hormonal alterations, persistent low-grade inflammatory response, mitochondrial impairment, and physical inactivity, all of which, as an ensemble, cause an imbalance between protein synthesis and breakdown in muscle tissue (Wu et al., 2023). Sarcopenia molecular mechanisms are complex and include age-related modification of diverse regulatory processes. Decreases of anabolic hormones, of which the most salient is testosterone, growth hormone, and insulin-like growth factor-1 (IGF-1), significantly disable regenerative capacity of the muscle and protein synthetic machine (Boccardi, 2024). At the same time, along with the progression of age, chronic inflammatory processes, as exemplified by heightened levels of pro-inflammatory cytokines TNF-α and IL-6, initiate proteolytic cascades, thereby triggering muscle protein breakdown (Shen et al., 2024). Recent transcriptomic accounts have characterised distinctive patterns of invasion of immume cells of sarcopenic muscle, including heightened numbers of pro-inflammatory T cells and macrophages, as being part of inflammatory microenvironment.

Functional impairment of mitochondria is the core of pathogenesis of sarcopenia, and is characterized by decreased oxidative capacity, enhanced formation of reactive oxygen species, and impaired mitochondrial biogenesis (Wu et al., 2023). Such muscle energetic impairments induce selective type II (fast-twitch) muscle fibre loss most susceptible to senescent atrophy. Moreover, structural-functional alterations of motor unit that accompany ageing, involving motor neuron loss and reorganisation of neuromuscular junction, induce further advancement of muscle weakness and decline of muscle performance.

### 2.2 Cachexia

Cachexia is a intense, multifactorial metabolic syndrome of muscle catabolism that is associated with involuntary weight loss, inflammatory body responses, and metabolic deregulation (Ebner et al., 2020). It can also be differentiated from simple starvation or senescent muscle loss because cachexia is present despite the presence of underlying chronic illnesses, most of which is cancer, but also heart failure, chronic kidney disease, chronic obstructive respiratory disease, and HIV/AIDS. It is present in 80% of terminal cancer patients and is directly responsible for decreased tolerance to treatment, decreased quality of life, and enhanced mortality (Dudhat et al., 2025; Ferrer et al., 2023; Setiawan et al., 2023).

Pathophysiology of cachexia involves diffuse perturbation of normal homeostasis of metabolism due to tumor products and host inflammatory response (Liu et al., 2024). Bloodborne cytokines, such as TNF-α, IL-1β, IL-6, and interferon-gamma, induce an environment of catabolism by up-regulating muscle proteolysis, lipolysis, and disrupting carbohydrate metabolism (Li X. et al., 2024). These inflammatory mediators activate the ubiquitin-proteasome system and autophagylysosome pathway while simultaneously suppressing the lGF-1/Pl3K/Akt/mTOR anabolicsignaling cascade, creating an environment that strongly favors muscle catabolism overanabolism. One of its special features is the occurrence of anorexia and derangement of energy metabolism that is not easily reversed by nutritional supplementation (Ebner et al., 2020). Products of tumor as well as inflammatory cytokines cause hypothalamic centers of inhibitions of appetite as well as enhanced resting energy expenditure, resulting into negative energy balance. Cachexia is also caused through the induction of insulin resistance, deranged lipid metabolism, as well as enhanced gluconeogenesis, each of these being solely responsible for depletion of muscle as well as fat tissue store.

### 2.3 Disuse atrophy

Disuseatrophy is the rapid, spontaneous muscle weakness and muscle reduction that occurs because of mechanical loading reduction as a consequence of physical inanition, immobilization, spaceflight, orbed rest (Boccardi, 2024). This type of muscleatrophy can occur at an arbitrary age and emphasizes the necessity of mechanical stimulation for the sustenance of muscle homeostasis. Disuseatrophy is very rapidly elicited, as off-loading leads to the reduction of muscle protein synthesis measurably within several hours, and extensive muscle mass is catabolized within days. They also include rapid downregulation of anabolic signaling, of which the most prominent is the IGF-1/PI3K/Akt/mTOR pathway, along with activation of proteolytic networks (Wu et al., 2023). Lower mechanical tension results in lowered mechanotransduction signaling, under other conditions maintaining basal protein synthetic levels as well as inhibiting proteolytic gene expression. Disuse also initiates upregulation of the E3 ubiquitin ligases MuRF1 and MAFbx/atrogin-1, main controllers of muscle protein breakdown by the ubiquitin-proteasome pathway.

Rapid mitochondrial adaptations take place upon disuse, and decreased mitochondrial content, decreased oxidative capacity, and enhanced reactive oxygen species formation have been implicated as contributing to muscle dysfunction (Boccardi, 2024). These changes particularly affect type I (slow-twitch) oxidative fibers, which rely heavily on mitochondrial metabolism. The preferential atrophy of type I fibers during disuse contrasts with the type II fiber vulnerability observed in sarcopenia, highlighting the specificity of atrophic responses to different stimuli (Wang and Pessin, 2013).

### 2.4 Neuromuscular disease-associated atrophy

Neuromuscular disorders encompass a diverse group of conditions affecting motor neurons, peripheral nerves, neuromuscular junctions, or muscle fibers themselves, all resulting in progressive muscle wasting and weakness (Abati et al., 2022). These include amyotrophic lateral sclerosis (ALS), spinal muscular atrophy (SMA), muscular dystrophies, and peripheral neuropathies. The muscle atrophy in these conditions results from denervation, impaired neuromuscular transmission, or primary myopathic processes, each presenting unique therapeutic challenges.

In denervation atrophy, loss of neural input triggers rapid muscle fiber atrophy through multiple mechanisms. The absence of neural activity eliminates trophic support normally provided by motor neurons, including neurotrophic factors and activity-dependent gene expression. Denervated muscle exhibits increased sensitivity to proteolytic stimuli, altered calcium handling, and mitochondrial dysfunction. The molecular signature of denervation includes upregulation of embryonic myosin heavy chains, acetylcholine receptor subunits, and specific atrophy-related genes distinct from those activated in disuse or systemic wasting conditions. Recent advances in understanding neuromuscular disease mechanisms have revealed the critical role of impaired protein homeostasis, oxidative stress, and inflammatory processes in disease progression (Abati et al., 2022). For instance, in ALS, motor neuron degeneration leads to progressive denervation, while inflammatory activation and metabolic dysfunction in muscle tissue contribute to the severe muscle wasting observed in advanced disease. Similarly, in muscular dystrophies, primary defects in structural proteins lead to mechanical fragility, chronic regeneration-degeneration cycles, and eventual replacement of muscle tissue with fibrotic and adipose tissue (Verma et al., 2022).

### 2.5 Overlapping and mixed etiologies

Clinicians most typically regard muscle wasting as the effect of multiple concurrent etiologies, but never as discrete diseases (Liu et al., 2024). An old carcinoma patient might concurrently have his/her sarcopenia due to his/her old age, cachexia due to his/her carcinoma, and disuse osteopathy due to restriction of physical movement as an adjunct of treatment. This multiplicity complicate diagnosis, prognosis, as well as planning of treatment because what is treatment enough for one is not for another. Interactions between different etiologies of muscle wasting have synergistic associations that cause acceleration of muscle loss that is greater than would ensue for each condition individually (Boccardi, 2024). These complex overlaps highlight the need for more refined preclinical models that capture multifactorial muscle wasting, a challenge recently emphasized in comparative analyses of animal models. For example, concomitance of cachexia of disease and sarcopenia of age results in exceptional universal muscle depletion since each of these conditions has classical proteolytic mechanisms that are analogous but other distinctive mechanisms. For example, hospitalization for an undue length of time in late age can cause disuse atrophy superimposed upon preexistent sarcopenia resulting in disastrous functional decline. Understanding these intersecting etiologies is critical for developing treatment plans that treat these processes holistically. This molecular crosstalk of disparate atrophic signals also suggests that combination therapies of multiple pathways could be necessary for optimum benefit (Lee et al., 2024). As one illustrative case, nutritional supplementation would perhaps not be enough for cachexia but could be of benefit as an adjunct to anti-inflammatory therapy and physical therapy that also corrects the disuse component. Acknowledgment of mixed etiologies is also equally imperative for bedside assessment and follow-up. Assessment strategies ought to remain general enough to acknowledge all of the causative variables for muscle wasting because treatment of only one but not other contributing variables is most likely to futile therapy (Li L. et al., 2024). This complexity also would dictate multidisciplinary follow-up teams and simultaneous treatment regimens for management of the complex process of muscle wasting under bedside conditions.

### 2.6 Sarcopenic obesity

Sarcopenic obesity (SO) is increasingly recognized as a distinct phenotype within the spectrum of muscle wasting disorders. It is defined by the coexistence of obesity—characterized by excess fat mass—and sarcopenia, the loss of skeletal muscle mass and function. The ESPEN–EASO consensus statement has provided a structured diagnostic framework for SO, recommending a two-stage process of screening and confirmatory diagnosis using combined functional and compositional criteria. The mechanisms underlying SO are complex and bidirectional. Adipose tissue dysfunction, particularly visceral fat accumulation, precipitates metabolic disturbances including low-grade inflammation, oxidative stress, insulin resistance, and hormonal dysregulation, all of which accelerate muscle catabolism. Conversely, reduced muscle mass and strength lower total energy expenditure, limit mobility, and promote further fat accumulation. This creates a vicious cycle of adipose–muscle interactions that drives disease progression. Moreover, myosteatosis—the ectopic infiltration of fat within muscle—is increasingly recognized as a hallmark lesion, negatively impacting contractility, metabolism, and insulin sensitivity. SO is associated with synergistically worse outcomes than either obesity or sarcopenia alone, including higher risks of frailty, disability, cardiometabolic disease, and mortality. Importantly, therapeutic weight loss in individuals with obesity, particularly through inadequate dietary supervision or after bariatric surgery, can aggravate muscle wasting if not paired with protective strategies such as resistance training and adequate protein intake. SO can manifest as primary (linked to age-related sarcopenia exacerbated by adiposity) or secondary (due to comorbid conditions accelerating both fat accumulation and muscle catabolism). The ESPEN–EASO consensus further recommends staging: Stage I, when muscle dysfunction and fat excess coexist without complications; and Stage II, when adverse health outcomes such as metabolic disease, physical disability, or cardiopulmonary compromise are present.

## 3 Pathophysiological mechanisms

### 3.1 Imbalance between protein synthesis and degradation

**FIGURE 2:**
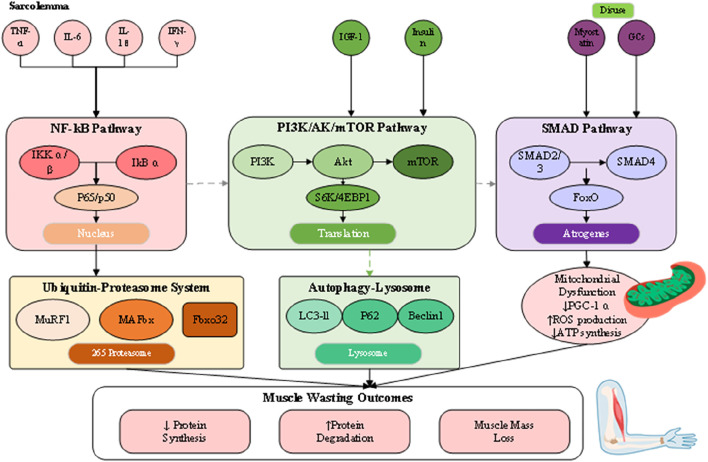
Integrated pathophysiological mechanisms of muscle wasting.

The pathophysiology of muscle loss reflects a disturbed equilibrium between protein synthesis and degradation, driven by interconnected molecular and cellular pathways that culminate in net protein depletion, blunted regenerative potential, and functional impairment (Wu et al., 2023). Although their relative contributions differ across sarcopenia, cachexia, and disuse atrophy, these processes converge on shared terminal pathways (Figure 2). Understanding this dynamic proteostatic balance is essential for identifying therapeutic targets and developing strategies that restore homeostasis.

Skeletal muscle mass is normally maintained through coordination between anabolic signaling and proteolytic pathways. Central to the anabolic axis is the insulin-like growth factor 1 (IGF-1)/phosphoinositide 3-kinase (PI3K)/Akt/mammalian target of rapamycin (mTOR) cascade (Boccardi, 2024). Activation of IGF-1 receptors promotes PI3K–Akt phosphorylation, leading to mTOR stimulation, phosphorylation of S6 kinase and 4EBP1, and the enhancement of mRNA translation and ribosome biogenesis. Under wasting conditions, this pathway is significantly repressed through reduced IGF-1 expression, insulin insensitivity, and direct inhibition by inflammatory cytokines or myostatin (Abati et al., 2022). Conversely, catabolic processes are primarily regulated by two proteolytic mechanisms: the ubiquitin-proteasome system (UPS) and the autophagy–lysosome system. The UPS selectively degrades short-lived or damaged myofibrillar proteins, with muscle-specific E3 ubiquitin ligases such as MuRF1 and MAFbx/atrogin-1 serving as critical regulators (Wetzlich et al., 2025). These atrogenes are transcriptionally controlled by FoxO proteins, which become activated upon Akt suppression, creating a molecular switch that simultaneously inhibits synthesis while promoting targeted degradation (Kramerova et al., 2020).

The autophagy–lysosome pathway, by contrast, contributes to organelle turnover, mitochondrial quality control, and clearance of larger protein aggregates. Its role is particularly evident in cachexia and disuse atrophy, where sustained activation—often mediated by inflammatory cytokines and oxidative stress—leads to excessive removal of mitochondria and sarcoplasmic constituents, exacerbating muscle weakness. Although autophagy is protective under physiological conditions of repair, chronic upregulation converts it into a maladaptive driver of wasting. Functional overlap exists between UPS and autophagy, as both converge on FoxO-dependent transcriptional programs, yet distinct regulatory checkpoints suggest that combined inhibition must be carefully titrated to avoid accumulation of dysfunctional proteins and organelles. Evidence from animal atrophy models further underscores these complexities, as accelerated rodent models commonly exaggerate UPS activation yet fail to replicate the gradual interplay of autophagy in human sarcopenia and cachexia, complicating direct translation of findings. Collectively, these insights provide a more comprehensive perspective on proteostatic regulation in muscle wasting, while also highlighting the challenges of targeting degradation pathways therapeutically.

### 3.2 Inflammatory cytokines and catabolic signaling

Chronic inflammation represents a central driver of muscle wasting, particularly in cachexia and age-related sarcopenia. Pro-inflammatory cytokines including tumor necrosis factor-alpha (TNF-α), interleukin-6 (IL-6), interleukin-1 beta (IL-1β), and interferon-gamma (IFN-γ) orchestrate a complex catabolic response through activation of specific intracellular signaling cascades. Recent transcriptomic analyses have revealed that these inflammatory mediators not only directly affect muscle cells but also reshape the muscle immune microenvironment, with infiltrating macrophages and T cells contributing to sustained inflammation and muscle damage (Shen et al., 2024). TNF-α, originally identified as cachexin for its role in cancer-associated wasting, activates the nuclear factor kappa-light-chain-enhancer of activated B cells (NF-κB) pathway through binding to TNF receptors on the muscle cell surface (Li X. et al., 2024). The canonical NF-κB signaling involves phosphorylation and degradation of inhibitor of κB (IκB) proteins by the IκB kinase (IKK) complex, allowing NF-κB dimers to translocate to the nucleus and induce transcription of numerous catabolic genes. This includes direct upregulation of MuRF1 expression and suppression of MyoD, a master regulator of muscle differentiation and regeneration. The NF-κB pathway also promotes oxidative stress and mitochondrial dysfunction, creating a feed-forward loop that amplifies muscle damage. IL-6 represents a pleiotropic cytokine with context-dependent effects on muscle metabolism. While acute, transient IL-6 elevation during exercise promotes beneficial adaptations, chronic IL-6 exposure in inflammatory conditions activates the Janus kinase/signal transducer and activator of transcription 3 (JAK/STAT3) pathway, leading to muscle wasting (Shen et al., 2024). STAT3 activation induces expression of suppressor of cytokine signaling 3 (SOCS3), which interferes with insulin and IGF-1 signaling, thereby contributing to anabolic resistance. Additionally, IL-6 stimulates the acute phase response in the liver, redirecting amino acids away from muscle protein synthesis toward production of acute phase proteins, further exacerbating muscle loss in systemic inflammatory conditions.

### 3.3 Mitochondrial dysfunction and oxidative stress

Mitochondrial dysfunction emerges as both a cause and consequence of muscle wasting, creating a vicious cycle that perpetuates muscle deterioration. In aging and disuse atrophy, reduced mitochondrial biogenesis occurs through downregulation of peroxisome proliferator-activated receptor gamma coactivator 1-alpha (PGC-1α), the master regulator of mitochondrial biogenesis. This leads to decreased mitochondrial content, impaired oxidative phosphorylation capacity, and reduced ATP production, compromising the energy-dependent processes required for maintaining muscle mass and function. The accumulation of dysfunctional mitochondria generates excessive reactive oxygen species (ROS), which serve as signaling molecules that activate proteolytic pathways (Wu et al., 2023). ROS activate redox-sensitive transcription factors including NF-κB and FoxO, directly linking oxidative stress to increased expression of atrogenes. Furthermore, oxidative damage to mitochondrial DNA and proteins impairs the electron transport chain, creating a self-perpetuating cycle of mitochondrial dysfunction and ROS production. This particularly affects type I oxidative fibers, which rely heavily on mitochondrial metabolism, explaining their preferential atrophy in disuse conditions. Recent evidence suggests that impaired mitochondrial quality control mechanisms, including mitophagy and mitochondrial dynamics, contribute significantly to muscle wasting (Boccardi, 2024). The accumulation of damaged mitochondria not only reduces cellular energy production but also triggers apoptotic signaling through release of cytochrome c and other pro-apoptotic factors. This mitochondrial-mediated cell death pathway represents an irreversible mechanism of muscle fiber loss, particularly relevant in severe wasting conditions where regenerative capacity is overwhelmed.

### 3.4 Hormonal dysregulation

Hormonal imbalances play a critical role in the pathogenesis of muscle wasting, with alterations in both anabolic and catabolic hormone levels contributing to net muscle protein loss. The age-related decline in anabolic hormones, including testosterone, growth hormone (GH), and insulin-like growth factor-1 (IGF-1), creates a permissive environment for muscle atrophy. Testosterone deficiency, prevalent in aging men and patients with chronic diseases, reduces muscle protein synthesis through both direct effects on androgen receptors in muscle and indirect effects through decreased IGF-1 production. The development of selective androgen receptor modulators (SARMs) represents an attempt to harness the anabolic effects of androgens while minimizing adverse effects (Fonseca et al., 2020). Insulin resistance, a hallmark of metabolic dysfunction in aging and chronic disease, severely impairs the muscle’s anabolic response to nutrients. Under normal conditions, insulin promotes muscle protein synthesis through activation of the PI3K/Akt/mTOR pathway while simultaneously suppressing proteolysis. In insulin-resistant states, this dual action is lost, resulting in reduced postprandial protein synthesis and sustained activation of proteolytic pathways even in the fed state. This anabolic resistance to insulin contributes significantly to the difficulty in maintaining muscle mass through nutritional interventions alone in conditions such as sarcopenia and cachexia. Cortisol and other glucocorticoids represent potent catabolic hormones that are often elevated in stress, chronic disease, and aging. Glucocorticoids directly induce expression of atrogenes including MuRF1 and MAFbx through activation of glucocorticoid receptors, while simultaneously suppressing IGF-1 signaling and inhibiting muscle satellite cell proliferation. The catabolic effects of glucocorticoids are particularly pronounced in type II muscle fibers, contributing to the preferential atrophy of these fibers observed in various wasting conditions. Additionally, glucocorticoids promote muscle insulin resistance, creating a synergistic catabolic environment when combined with inflammatory cytokines. Myostatin, a member of the transforming growth factor-beta (TGF-β) superfamily, functions as a powerful negative regulator of muscle mass (Abati et al., 2022; Wetzlich et al., 2025). Binding of myostatin to activin type II receptors triggers SMAD2/3 phosphorylation and subsequent activation of FoxO transcription factors, leading to increased expression of atrogenes and suppression of Akt signaling. Myostatin levels are elevated in multiple muscle wasting conditions, making it an attractive therapeutic target. However, clinical trials of myostatin inhibitors have shown mixed results, with increases in muscle mass not always translating to functional improvements (Wetzlich et al., 2025; Kramerova et al., 2020). This highlights the complexity of muscle regulation and the need for combination approaches targeting multiple pathways.

### 3.5 Neural and neuromuscular factors

**FIGURE 3:**
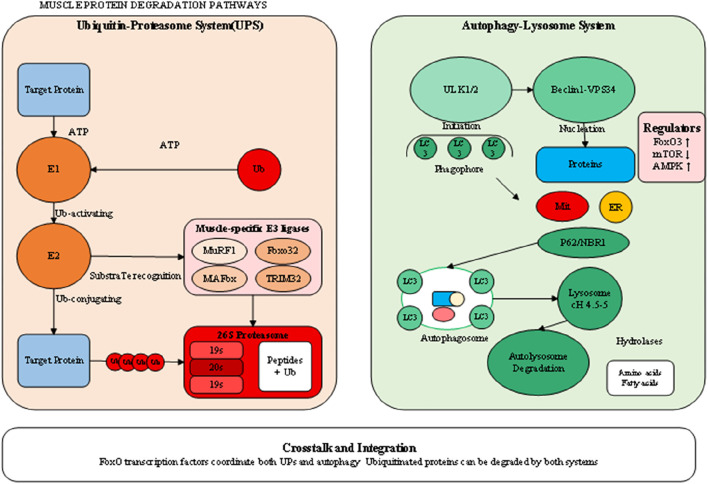
Molecular mechanisms of protein degradation systems in muscle wasting.

The neuromuscular system plays a fundamental role in maintaining muscle mass and function, with disruptions at any level from motor neurons to neuromuscular junctions contributing to muscle wasting (Abati et al., 2022). Age-related loss of motor neurons, particularly those innervating type II muscle fibers, results in motor unit remodeling and fiber type grouping characteristic of sarcopenia. The surviving motor units attempt to reinnervate denervated fibers, but this compensatory mechanism becomes overwhelmed with advancing age, leading to irreversible fiber loss. Recent evidence suggests that this motor neuron loss may be driven by oxidative stress, mitochondrial dysfunction, and neuroinflammation within the spinal cord. NMJ integrity is required for effective neural command of muscle contraction, and structural and functional alterations of the NMJ are essential for muscle weakness as a consequence of age and disease (Kramerova et al., 2020). Age-related changes include postsynaptic disintegration of the machinery, reduction of acetylcholine receptor density, as well as defective synaptic conduction. These alterations are exacerbated by inflammatory cytokines and oxidative stress, creating an environment that is deleterious for NMJ maintenance. Deficiency of agrin-MuSK signaling, which is required for maintenance of stability of the NMJ, also is culpable for acceleration of additional synaptic loss and concomitant muscle fiber atrophy. Denervation is the most potent of neural input into muscle atrophication and is characterized by conditions like amyotrophic lateral sclerosis, spinal muscular atrophy, and peripheral neuropathies (Kramerova et al., 2020). Reduction of neural input leads into quick reprogramming of muscle gene expression, for instance, upregulation of acetylcholine receptors, voltage-gated sodium channels, and embryonic myosin heavy chains. Denerved muscles are extremely susceptible to proteolytic challenge and exhibit quick, intervention-insensitive atrophication. Denervation is characterized by activation of HDAC4 and myogenin that cause an unique transcription profile leading into muscle fibre atrophication and further deterioration. Detailed molecular events of protein degrading processes are depicted in Figure 3, demonstrating the complex regulation of the ubiquitin-proteasome system and autophagy-lysosome pathway, where shared regulatory mechanisms are present. There is an imperative for an understanding of these complex communications for designing selective therapeutic intervention, capable of modulating protein breakdown effectively but maintaining needed processes of cells. This type of coordination of these processes, as induced by transcription factors like FoxO proteins, is necessary for the efficient breakdown of damaged protein but can also cause excessive breakdown of muscle proteins under pathological conditions. Briefly, muscle wasting pathophysiology is an unified collection of associated processes, collectively that push the balance toward net protein loss. All these processes, as they exist, have no independent functioning but pervasive crosstalk and reciprocal activation, complicating therapeutic intervention. Effective treatment regimens would, as corollary, most likely involve simultaneous targeting of several of these paths, concomitantly addressing as far as possible the anabolic signaling inhibition and catabolic process activation that define the muscle wasting diseases.

Despite significant progress, several unresolved issues remain in understanding the molecular mechanisms of muscle wasting. Animal models often fail to replicate the chronic, multifactorial progression of human disease, limiting translational relevance. Similar concerns have been articulated in critiques of muscle atrophy models, particularly regarding the discrepancy between accelerated rodent models and the slower, cumulative trajectory seen in humans. Moreover, while pathways such as IGF-1/Akt/mTOR, myostatin signaling, and inflammatory cascades have been characterized, controversies persist regarding the relative weight of each in different conditions (e.g., sarcopenia vs. cachexia). The paradox of increased muscle mass without proportional functional improvement in myostatin inhibition trials further highlights our incomplete understanding of muscle “quality” versus “quantity.” Future work must focus on integrating molecular, metabolic, and neuromuscular perspectives to clarify these discrepancies.

## 4 Diagnostic approaches

### 4.1 Clinical criteria and definitions

Accurate diagnosis of muscle wasting involves an integrated, multimodal process that brings together clinical evaluation, tests of functionality, sophisticated imaging modalities, as well as newly emerging biomarkers to surmount the complex nature of muscle loss as well as dysfunction (Li L. et al., 2024). Considering the diverse etiology as well as variable presentation of muscle wasting conditions, the diagnosis techniques should have adequate sensitivity for differentiation of early alterations as well as have special consideration for the simultaneous pathophysiology. Advances of imaging technology as well as biomarker identification have greatly facilitated diagnosis as well as follow-up of muscle wasting, but there are still concerns as regards standardization of diagnosis, in various populations as well as various health facilities. The diagnosis of muscle wasting is based on various complementing techniques providing a comprehensive assessment of multiple aspects of muscle health, requiring integration of clinical, functional, and biochemical approaches. Standardized diagnosis is also among the foremost challenges of muscle wasting disorders, as various divergent definitions have emerged internationally that take into consideration the heterogeneity of conditions. It has also evolved to initiate an algorithm of diagnosis put forth by the European Working Group on Sarcopenia in Older People (EWGSOP2), that starts first considering muscle strength as its first parameter, validated by documentation of reduction of muscle quantity or quality, severity being indicated by physical performance measures (Sayer et al., 2024). This shifting of focus from muscle mass to muscle function is an admission of broader understanding that weakness is ever antecedent and surviving antecedent loss of mass, allowing diagnosis earlier and better concordance of diagnosis and outcomes. They have also calibrated these criteria such that ethnic group difference of body composition is allowed for, setting population-specific cut-off values for handgrip strength (<28 kg for men, <18 kg for women), for walking speed (<1.0 m/s), and for skeletal muscle mass index (Chen et al., 2020). These localized adjustmentssuggest the necessity for consideration of anthropometric difference for application of criteria for diagnosis across populations. For cachexia, cross-national agreed definition is >5% weight loss within 12 months or BMI<20 kg/m^2^with three of five of the following: decline of muscle strength, weakness, anorexia, decreased fat-free mass index, or deranged biochemistry, including inflammatory markers (Ebner et al., 2020).

### 4.2 Anthropometric and functional assessments

Functional tests provide easy, inexpensive screening assessments that can be quickly administered in clinical settings, serving as the foundation for muscle wasting diagnosis. Handgrip strength on calibrated dynamometry is an inexpensive but potent general index of muscle function that correlates strongly to strength of the lower limb and has predictive validity for adverse outcomes such as disability, hospital admission, and mortality (Li L. et al., 2024). There is a three-reading-per-hand protocol, where the highest reading is reported, but hand dominance, presence of arthritis, and acute sickness must be adjusted for for interpretation. Gait speed measured for 4–6 m yields an objective physical performance measure, for which speeds of 0.8 m or lower signify intense sarcopenia and heightened risk of falls (Chen et al., 2020). Supplemental measures of function, used together as the five-time sit-to-stand measure, Timed Up and Go (TUG) measure, and Short Physical Performance Battery (SPPB) allow for detailed characterization of function. These measures signify disparate aspects of muscle function, ranging from power generation as employed for movement of chairs to assessment of dynamic balance as measured by TUG testing, upon which identification of diverse impairments of function can direct selective intervention.

### 4.3 Advanced imaging techniques

Modern imaging techniques have transformed muscle quantity and quality assessment possibilities, allowing for quantitative determination of muscle mass, as well as registering of pathological alterations such as fat infiltration and fibrosis (Chianca et al., 2022). Dual-energy X-ray absorptiometry (DEXA) is still most preferred method of estimation of appendicular lean mass because of ease of use, minimal use of radiation, and available reference databases. Appendicular skeletal muscle mass index (ASMI = ALM/height^2^) determination allows body-size adjusted value for diagnosis of lowered muscle mass, but DEXA is incapable of discriminating between muscle and other lean tissues, and analyzing muscle quality (Li L. et al., 2024). Computed tomography (CT) and magnetic resonance imaging (MRI) are still the gold standards for muscle evaluation, being precise for muscle cross-sectional area, volume, and composition values (Salaffi et al., 2022). L3 (third lumbar vertebra) level CT scans have gained unprecedented popularity among oncology centers for being used for the purpose of ascertaining sarcopenia and treatment toxicity evaluation. Muscle radiodensity can even be quantitated, enabling estimation of the existence of myosteatosis, an antecedent precursor of the onset of muscle dysfunction that typically occurs antecedent to mass loss. MRI offers similar capabilities without radiation exposure, with advanced sequences enabling assessment of muscle perfusion, metabolism, and inflammatory changes (Messina et al., 2018). Ultrasound has emerged as a valuable point-of-care tool for muscle assessment, particularly in critically ill or immobile patients where other imaging modalities are impractical (Salaffi et al., 2022). Measurement of muscle thickness, cross-sectional area, and echogenicity provides real-time assessment of muscle quantity and quality. The pennation angle and fascicle length measurements offer insights into muscle architecture changes that affect force-generating capacity. However, ultrasound remains operator-dependent, requiring standardized protocols and training to ensure reproducibility. Recent developments in automated image analysis using artificial intelligence show promise for improving reliability and accessibility of ultrasound-based muscle assessment.

### 4.4 Biochemical and molecular biomarkers

**FIGURE 4:**
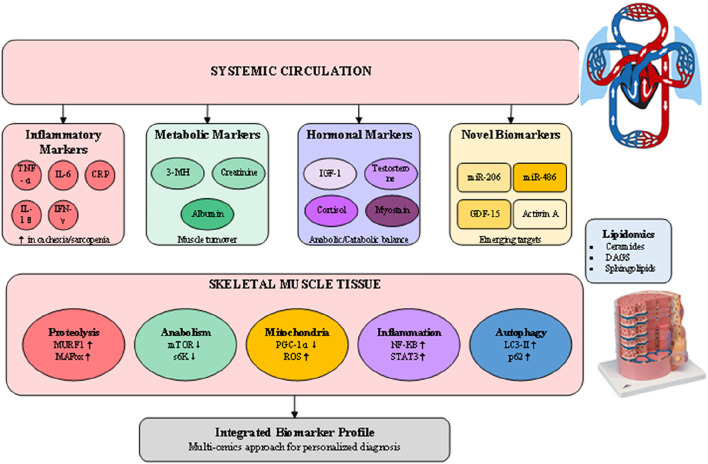
Molecular biomarkersand pathways in muscle wasting diagnosis.

The development of reliable biomarkers for muscle wasting represents a critical advancement in diagnostic capabilities, offering objective measures that complement clinical and imaging assessments (Hsu et al., 2024). As illustrated in Figure 4, the biomarker landscape encompasses traditional inflammatory markers, metabolic indicators, hormonal profiles, and emerging molecular signatures that collectively provide insights into the underlying pathophysiology of muscle loss. These circulating biomarkers not only aid in diagnosis but also offer potential for monitoring disease progression and treatment response. Traditional inflammatory markers including C-reactive protein (CRP), tumor necrosis factor-alpha (TNF-α), and interleukin-6 (IL-6) remain valuable indicators of the systemic inflammation driving muscle wasting, particularly in cachexia (Han et al., 2024). Elevated IL-6 levels correlate with muscle mass loss and functional decline in both cancer cachexia and age-related sarcopenia, though the overlap with other inflammatory conditions limits specificity. The IL-6/albumin ratio has emerged as a more specific marker, integrating inflammatory status with nutritional assessment to improve diagnostic accuracy in cancer-associated muscle wasting. Metabolic biomarkers provide direct evidence of muscle protein turnover and metabolic dysfunction. Urinary 3-methylhistidine excretion reflects muscle protein breakdown rates, though dietary protein intake must be controlled for accurate interpretation. Novel metabolomic approaches have identified specific lipid signatures associated with sarcopenia, including elevated ceramides and diacylglycerols that indicate lipotoxicity and insulin resistance in muscle tissue. Recent lipidomic profiling studies have revealed distinct patterns of circulating sphingolipids and phospholipids that differentiate sarcopenic from healthy older adults, offering potential for early detection before significant muscle loss occurs (Hsu et al., 2024). The emergence of microRNAs (miRNAs) as biomarkers represents a paradigm shift in muscle wasting diagnostics (Han et al., 2024; Qaisar et al., 2021). Muscle-specific miRNAs (myomiRs) including miR-206, miR-1, and miR-133a are released into circulation during muscle damage and regeneration, providing sensitive indicators of muscle health. The ratio of miR-206 to miR-1 appears particularly promising for distinguishing between different muscle wasting etiologies, with elevated ratios indicating impaired regenerative capacity. Additionally, miR-486 downregulation correlates with activation of proteolytic pathways and serves as an early marker of anabolic resistance in aging muscle. Another promising domain is metabolomics, which has begun to reveal circulating metabolites linked to muscle decline.

### 4.5 Integrated diagnostic approaches

The complexity of muscle wasting necessitates integrated diagnostic strategies that combine multiple assessment modalities to capture the multifaceted nature of these conditions (Li L. et al., 2024). Current guidelines recommend a tiered diagnostic approach, beginning with clinical screening and progressing through functional, biochemical, and imaging assessments based on initial findings. This systematic approach ensures efficient resource utilization while maintaining diagnostic accuracy through the integration of multiple complementary modalities. Composite diagnostic tools such as the SARC-F questionnaire provide rapid screening in primary care settings, though low sensitivity necessitates confirmation with objective measures (Chen et al., 2020). The addition of calf circumference to create SARC-CalF improves sensitivity without sacrificing specificity, offering a simple enhancement for community-based screening programs. Machine learning algorithms integrating clinical, functional, and biomarker data show promise for improving diagnostic accuracy and predicting individual risk trajectories, though validation in diverse populations remains necessary. The integration of multi-omics approaches combining genomics, proteomics, metabolomics, and lipidomics offers unprecedented insights into muscle wasting pathophysiology (Yang et al., 2024). These comprehensive molecular profiles enable identification of distinct phenotypes within broad diagnostic categories, facilitating personalized therapeutic approaches. For instance, metabolomic profiling can distinguish between inflammation-driven and disuse-related muscle loss, guiding selection of anti-inflammatory versus exercise-based interventions. The development of point-of-care devices incorporating multiple biomarker measurements promises to bring sophisticated diagnostics to clinical practice.

### 4.6 Challenges and future directions in diagnosis

Despite significant advances, several challenges persist in muscle wasting diagnosis (Li L. et al., 2024; Tsai, 2024). The lack of universally accepted diagnostic criteria across different conditions and populations creates confusion in clinical practice and limits comparability between studies. Ethnic variations in body composition, muscle strength norms, and biomarker levels necessitate population-specific reference values that are not yet available for many groups. The dynamic nature of muscle wasting, with fluctuating inflammatory status and variable progression rates, requires repeated assessments that may not be feasible in all healthcare settings. Emerging technologies offer solutions to current diagnostic limitations (Lai et al., 2024). Integration of improved animal/cell models alongside advanced diagnostics is vital, as recent reviews have noted that oversimplified atrophy models can obscure biomarker validation. Artificial intelligence-based image analysis can standardize interpretation of ultrasound and cross-sectional imaging, reducing operator dependence and improving accessibility. Wearable devices continuously monitoring physical activity, muscle activation patterns, and metabolic parameters provide real-time data on functional capacity and early detection of deterioration. The integration of digital biomarkers from smartphones and activity trackers with traditional assessments creates comprehensive patient profiles that capture both capacity and performance in real-world settings. The future of muscle wasting diagnosis lies in precision medicine approaches that integrate multiple data streams to provide individualized risk assessment and guide targeted interventions (Liu et al., 2024; Zhang et al., 2021). Development of rapid, multiplexed biomarker panels suitable for point-of-care testing will enable routine screening in primary care settings. Longitudinal studies establishing trajectories of change in imaging and biomarker parameters will improve prognostic accuracy and define therapeutic windows for different interventions. The ultimate goal remains early detection of muscle wasting before irreversible changes occur, enabling preventive strategies that maintain muscle health throughout the lifespan.

Although diagnostic tools have advanced, major challenges hinder their clinical utility. Persistent heterogeneity in diagnostic criteria across working groups (EWGSOP2, AWGS) limits comparability of prevalence estimates and clinical implementation. Biomarkers such as IL-6 or microRNAs show promise but suffer from poor specificity due to overlap with other chronic conditions. Imaging modalities, while accurate, remain costly and impractical for routine use in many healthcare systems. A key controversy is whether diagnosis should prioritize muscle mass, muscle strength, or a composite from multi-omics data. Thus, efforts toward consensus, population-specific thresholds, and affordable point-of-care tools remain urgently needed.

## 5 Emerging therapeutic strategies

### 5.1 Nutritional interventions

The management of muscle wasting has evolved significantly beyond traditional supportive care, with emerging therapeutic strategies targeting specific molecular pathways, enhancing regenerative capacity, and addressing the multifactorial nature of muscle loss (Liu et al., 2024). These innovative approaches range from targeted nutritional interventions and novel pharmacological agents to advanced rehabilitation techniques and regenerative medicine, each offering unique mechanisms to restore the balance between muscle protein synthesis and degradation. The integration of these strategies into comprehensive treatment protocols represents a paradigm shift from symptom management to disease modification, though significant challenges remain in translating promising preclinical findings into clinical efficacy (Tsai, 2024). The therapeutic landscape for muscle wasting encompasses multiple complementary approaches that target different aspects of muscle metabolism and function. Nutritional strategies represent the foundation of muscle wasting management, with targeted supplementation addressing specific metabolic deficits while supporting overall anabolic processes (Beaudry et al., 2022). Nutritional interventions target multiple pathways including mTOR activation, inflammatory modulation, and mitochondrial support. The evolution from simple protein supplementation to sophisticated nutrient combinations reflects growing understanding of the complex nutritional requirements for maintaining muscle health in the face of catabolic challenges. Leucine, a branched-chain amino acid, functions as a potent activator of mTOR signaling independent of insulin, making it particularly valuable in insulin-resistant states common in sarcopenia and cachexia (Beaudry et al., 2022). Optimal leucine dosing requires 2.5–3 g per meal to overcome anabolic resistance in older adults, with studies demonstrating enhanced muscle protein synthesis when combined with resistance exercise. β-Hydroxy β-methylbutyrate (HMB), a leucine metabolite, provides additional benefits through inhibition of proteolytic pathways and reduction of muscle damage markers. Clinical trials have shown that HMB supplementation at 3 g daily can attenuate muscle loss during bed rest and improve recovery following hospitalization, though effects in ambulatory populations remain modest (Jang et al., 2021). Vitamin D supplementation addresses a critical deficiency prevalent in muscle wasting populations, with receptors present throughout muscle tissue influencing protein synthesis, mitochondrial function, and calcium handling. The optimal vitamin D status for muscle health appears to be 30–50 ng/mL 25-hydroxyvitamin D, with supplementation doses of 800–2000 IU daily required to achieve these levels in deficient individuals. Combined vitamin D and protein supplementation shows synergistic effects on muscle strength and physical performance, particularly in sarcopenic older adults with baseline deficiency. Omega-3 fatty acids, particularly eicosapentaenoic acid (EPA) and docosahexaenoic acid (DHA), exert anti-inflammatory effects while enhancing the muscle protein synthetic response to amino acids (Beaudry et al., 2022). Doses of 2–4 g daily of EPA + DHA have demonstrated efficacy in attenuating muscle loss in cancer cachexia and improving muscle quality in sarcopenia. The mechanisms involve suppression of NF-κB signaling, reduced expression of proteolytic genes, and enhanced insulin sensitivity. Recent evidence suggests omega-3 supplementation may also preserve mitochondrial function and reduce oxidative stress, contributing to improved muscle energetics.

### 5.2 Pharmacological agents

The pharmacological landscape for muscle wasting has expanded dramatically with the development of targeted agents addressing specific molecular pathways, though translation to clinical practice remains challenging (Tsai, 2024; Fonseca et al., 2020). Selective androgen receptor modulators (SARMs) represent an attempt to harness the anabolic effects of testosterone while minimizing androgenic side effects. Enobosarm (GTx-024) has shown promise in phase II trials, increasing lean body mass by 1–1.5 kg over 12–16 weeks in cancer patients, though phase III trials failed to meet functional endpoints (Fonseca et al., 2020). The disconnect between mass gains and functional improvements highlights the complexity of muscle regulation and the need for combination approaches. Myostatin inhibition remains an attractive therapeutic target given its role as a negative regulator of muscle mass (Wetzlich et al., 2025). Multiple approaches including monoclonal antibodies (bimagrumab), soluble receptors (ACE-031), and follistatin gene therapy have been investigated. While these agents consistently increase muscle mass, often dramatically, the translation to improved strength and function has been disappointing (Tsai, 2024). The compensatory activation of other TGF-β family members and the importance of myostatin in tendon homeostasis may explain these limitations. Current research focuses on combination strategies that pair myostatin inhibition with exercise or other anabolic interventions. Anti-inflammatory therapies targeting specific cytokines show promise in cachexia management, though systemic immunosuppression remains a concern (Tsai, 2024). Tocilizumab, an IL-6 receptor antibody, has demonstrated efficacy in maintaining muscle mass in rheumatoid arthritis patients, with ongoing trials in cancer cachexia. JAK inhibitors offer an alternative approach by blocking intracellular inflammatory signaling, potentially preserving beneficial cytokine functions while inhibiting muscle catabolism. The optimal timing and patient selection for anti-inflammatory interventions remain critical questions requiring further investigation. Emerging metabolic modulators target mitochondrial function and cellular energy homeostasis (Fonseca et al., 2020). Mitochondrial-targeted antioxidants such as MitoQ show promise in preclinical models, reducing oxidative damage while preserving mitochondrial biogenesis. AMPK activators including metformin and AICAR enhance mitochondrial function and may sensitize muscle to anabolic stimuli, though concerns about interference with exercise adaptations require careful consideration. The development of tissue-specific metabolic modulators represents a priority for avoiding systemic metabolic effects.

### 5.3 Exercise and rehabilitation

Exercise remains the most potent stimulus for muscle adaptation, with different modalities offering complementary benefits for muscle wasting management. Resistance training specifically targets muscle mass and strength through mechanical loading that activates mTOR signaling, suppresses proteolytic gene expression, and stimulates satellite cell proliferation. Progressive resistance training programs achieving 70%–80% of one-repetition maximum for 2-3 sessions weekly can increase muscle mass by 1–2 kg and strength by 20%–30% even in frail older adults (Shen et al., 2023). The anabolic effects of resistance training are enhanced when combined with protein supplementation, particularly leucine-enriched formulations consumed within the post-exercise window. Aerobic exercise provides distinct benefits including enhanced mitochondrial biogenesis, improved insulin sensitivity, and reduced systemic inflammation (de Sousa Fernandes et al., 2020). Moderate-intensity continuous training at 60%–70% of maximum heart rate for 150 min weekly meets current guidelines, though high-intensity interval training may provide superior adaptations in time-efficient protocols. The molecular adaptations to aerobic exercise include PGC-1α activation, enhanced oxidative enzyme expression, and improved capillarization, collectively improving muscle quality independent of mass changes (Rodríguez-Gutiérrez et al., 2023). Combined training programs integrating resistance and aerobic components offer comprehensive benefits addressing multiple aspects of muscle health (Shen et al., 2023). The molecular interference between concurrent training modalities can be minimized through appropriate periodization, with resistance training preceding aerobic exercise in the same session or alternating training days. Multicomponent programs incorporating balance and flexibility training provide additional functional benefits, particularly important for fall prevention in sarcopenic individuals (Gomez-Pinilla and Thapak, 2024). Neuromuscular electrical stimulation (NMES) provides an alternative for patients unable to participate in voluntary exercise due to critical illness, severe deconditioning, or neurological impairment (Shen et al., 2023). High-frequency stimulation (50–100 Hz) can generate tetanic contractions sufficient to maintain muscle mass and even induce hypertrophy when applied at adequate intensity. The molecular responses to NMES include mTOR activation and suppression of proteolytic pathways, though the pattern differs from voluntary contractions. Integration of NMES with nutritional support and early mobilization in ICU settings has shown promise for preventing ICU-acquired weakness.

### 5.4 Regenerative and molecular approaches

**FIGURE 5:**
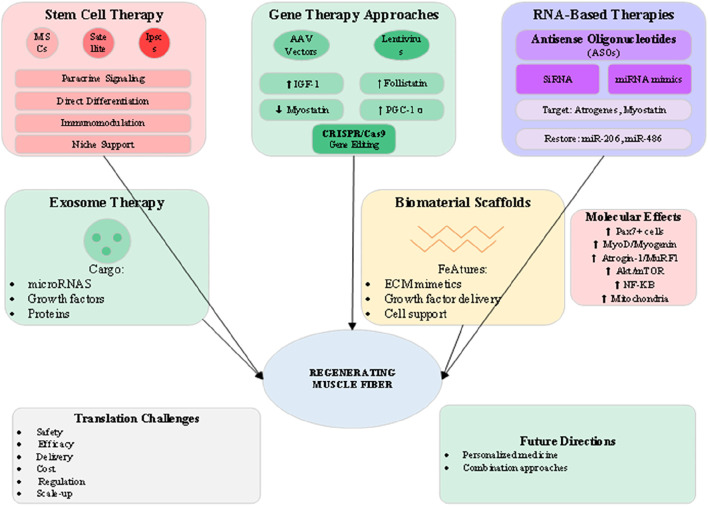
Regenerative medicine and advanced therapeutic approaches for muscle wasting.

The frontier of muscle wasting therapy lies in regenerative medicine approaches that aim to restore muscle tissue through cellular replacement, genetic modification, or activation of endogenous repair mechanisms (Chung et al., 2025). As illustrated in Figure 5, these advanced strategies encompass stem cell therapy, gene editing, RNA-based interventions, and tissue engineering approaches that collectively offer potential for disease modification rather than symptomatic management. The translation of these technologies from preclinical success to clinical application represents both tremendous opportunity and significant challenges. Stem cell therapy for muscle wasting focuses primarily on mesenchymal stem cells (MSCs) and muscle satellite cells, each offering distinct mechanisms of action (Chung et al., 2025). MSCs exert predominantly paracrine effects, secreting growth factors, cytokines, and extracellular vesicles that modulate inflammation, enhance angiogenesis, and support endogenous muscle regeneration. Clinical trials of systemic or intramuscular MSC delivery have shown modest benefits in muscle mass and function, though optimal cell sources, doses, and delivery methods remain under investigation. The immunomodulatory properties of MSCs may be particularly valuable in inflammatory muscle wasting conditions such as cachexia (Cai et al., 2022). Satellite cell transplantation aims to directly replenish the muscle stem cell pool depleted in chronic wasting conditions. However, challenges including poor cell survival, limited migration, and rapid differentiation have limited clinical success. Strategies to enhance satellite cell function include genetic modification to improve survival, preconditioning with pharmacological agents, and co-transplantation with supporting cell types. The development of induced pluripotent stem cell (iPSC)-derived myogenic progenitors offers a potentially unlimited cell source, though concerns about tumorigenicity and immune rejection require careful consideration. Gene therapy approaches targeting muscle wasting have advanced significantly with the development of adeno-associated virus (AAV) vectors showing muscle tropism (Lee et al., 2024). Overexpression of IGF-1 through AAV delivery promotes muscle hypertrophy and enhances regeneration while suppressing proteolytic pathways. Follistatin gene therapy, which inhibits multiple TGF-β family members including myostatin, has shown dramatic effects on muscle mass in preclinical models and early clinical trials. The development of muscle-specific AAV serotypes and regulatable expression systems addresses safety concerns while improving therapeutic efficacy (Qazi et al., 2019). RNA-based therapies offer precise modulation of gene expression without permanent genetic modification (Qazi et al., 2019). Antisense oligonucleotides (ASOs) targeting myostatin or atrogene expression have shown promise in preclinical models, with the success of ASOs in spinal muscular atrophy providing a clinical precedent. Small interfering RNA (siRNA) and microRNA mimics or inhibitors offer additional tools for modulating multiple pathways simultaneously. The delivery challenges for RNA therapeutics are being addressed through chemical modifications, nanoparticle formulations, and conjugation strategies that enhance muscle uptake and stability. Exosome therapy represents an emerging approach that harnesses the regenerative potential of extracellular vesicles (Cai et al., 2022). Muscle-derived exosomes contain specific cargo including myomiRs, growth factors, and signaling proteins that promote myogenesis and suppress inflammation. Engineering exosomes for enhanced therapeutic payload or targeting represents an active area of investigation. The advantages of exosome therapy include low immunogenicity, ability to cross biological barriers, and potential for repeated administration without loss of efficacy.

### 5.5 Combination strategies and personalized approaches

The complexity of muscle wasting pathophysiology necessitates combination therapeutic strategies that address multiple mechanisms simultaneously (Liu et al., 2024). The integration of nutritional, pharmacological, exercise, and regenerative interventions creates synergistic effects exceeding those achievable with monotherapy. The optimal combinations vary based on the underlying etiology, disease stage, and individual patient characteristics, highlighting the need for personalized treatment algorithms. The combination of resistance exercise with protein supplementation represents the most established multimodal approach, with meta-analyses demonstrating superior outcomes compared to either intervention alone (Shen et al., 2023). The timing of protein intake relative to exercise, the specific amino acid composition, and the exercise intensity all influence the magnitude of benefit. Addition of vitamin D to exercise and protein further enhances outcomes in deficient populations, while omega-3 fatty acids may augment the anabolic response to exercise through anti-inflammatory mechanisms (Beaudry et al., 2022). Pharmacological agents show enhanced efficacy when combined with exercise interventions (Fonseca et al., 2020). The mechanical stimulus of exercise may sensitize muscle to anabolic agents while the pharmacological support enables greater training intensity and recovery. For example, testosterone replacement combined with resistance training produces greater gains in muscle mass and strength than either intervention alone, though careful monitoring for adverse effects remains essential. Similarly, myostatin inhibition may be most effective when combined with exercise to ensure functional translation of increased muscle mass. The integration of regenerative approaches with conventional therapies offers exciting possibilities for enhanced therapeutic outcomes (Chung et al., 2025). Stem cell therapy combined with exercise rehabilitation may improve cell engraftment and differentiation while the exercise stimulus enhances the regenerative microenvironment. Gene therapy approaches targeting multiple pathways simultaneously, such as combined IGF-1 overexpression with myostatin inhibition, show promise for addressing the complex molecular alterations in muscle wasting.

### 5.6 Challenges and future perspectives

Despite remarkable advances in therapeutic development, significant challenges remain in translating promising interventions to clinical practice (Tsai, 2024). The heterogeneity of muscle wasting conditions, with varying contributions of inflammation, disuse, hormonal deficits, and neural factors, complicates the development of broadly effective therapies. Patient-specific factors including age, comorbidities, genetic background, and disease stage influence treatment response, necessitating personalized approaches guided by biomarker profiles and functional assessments. The disconnect between improvements in muscle mass and functional outcomes observed in many clinical trials highlights the complexity of muscle quality versus quantity (Wetzlich et al., 2025). Future therapeutic development must prioritize functional endpoints and patient-reported outcomes rather than focusing solely on muscle mass. The development of composite outcome measures incorporating strength, endurance, and daily function better captures clinically meaningful benefits. Additionally, the optimal timing of interventions relative to disease trajectory requires further investigation, with preventive strategies potentially offering greater benefit than treatment of established muscle wasting. Safety considerations remain paramount, particularly for interventions targeting fundamental cellular processes (Fonseca et al., 2020). Long-term effects of myostatin inhibition on tendon integrity, cardiac muscle, and metabolic homeostasis require careful evaluation. The potential for off-target effects of gene therapy, immunogenicity of biological agents, and metabolic consequences of dramatic muscle mass increases necessitate comprehensive safety monitoring. The development of tissue-specific delivery systems and controllable expression platforms addresses some safety concerns while improving therapeutic windows. The economic considerations of advanced therapies pose additional challenges for widespread implementation. Cell therapies, gene therapies, and biological agents carry substantial costs that may limit accessibility. Development of cost-effective production methods, demonstration of long-term cost savings through prevention of disability, and innovative reimbursement models will be essential for ensuring equitable access to emerging therapies. The integration of digital health technologies for remote monitoring and personalized exercise prescription offers potential for scalable, cost-effective interventions. Looking forward, the field of muscle wasting therapeutics stands at an inflection point where convergence of molecular understanding, technological capabilities, and clinical need creates unprecedented opportunities (Qazi et al., 2019). The development of multi-target drugs, engineered cell therapies, and precision medicine approaches guided by comprehensive molecular profiling promises to transform patient outcomes. Success will require continued investment in basic and translational research, innovative clinical trial designs that capture the complexity of muscle wasting, and multidisciplinary collaboration spanning molecular biology, bioengineering, clinical medicine, and rehabilitation science. The ultimate goal remains restoration of not just muscle mass but functional capacity and quality of life for the millions affected by muscle wasting disorders.

Therapeutic development is marked by both exciting innovations and persistent shortcomings. Pharmacological interventions, including SARMs and myostatin inhibitors, have repeatedly shown discordance between preclinical success and clinical trial outcomes, raising concerns about target specificity and off-target effects. Exercise remains the most effective intervention, but controversies persist regarding optimal modality, intensity, and feasibility in frail populations. Nutritional approaches demonstrate synergistic effects yet face uncertainty about long-term sustainability. Regenerative therapies such as stem cell and gene-based treatments hold promise but continue to confront safety, cost, and regulatory challenges. These limitations underscore the need for combination strategies and individualized treatment regimens rather than reliance on single-pathway solutions.

## 6 Limitations and future directions

### 6.1 Current challenges in diagnosis and treatment

The field of muscle wasting faces multifaceted challenges that significantly impede progress from basic science discoveries to improved patient outcomes. Diagnostic incongruences are literally a roadblock, since absence of universally agreed criteria between divergent muscle wasting diseases leads to misunderstanding between applications for research as well as for use under clinical practice (Li L. et al., 2024). Whilst EWGSOP2 and AWGS have brought diagnosis of sarcopenia to virtual standardization, however, there still remains significant variability between cutoff values, measures, as well as diagnosis algorithms (Sayer et al., 2024). Such variability results into very divergent prevalence estimates, renders cross-study comparisons of limited use, as well as ultimately translates into late relevant intervention for affected individuals. Underdiagnosis of muscle wasting still remains rampant, as there still continue to exist reports that below 1% of cases of sarcopenia receive formal diagnosis under clinical practice even as they occur among 10%–20% of aged individuals (Chen et al., 2020). Converting encouraging preclinical outcomes into clinical success is amongst the most formidable challenges of muscle wasting therapeutics (Tsai, 2024). Despite numerous hundreds of agents having startling outcomes within animals, most have failed to become capable of showing clinically substantial benefit under conditions of human trials. This translation breakdown is because of built-in model animal/human ailment distinction, for instance, species-specificity of muscle biology, abstracted nature of animal ailment induction as compared to complex multifactorial nature of human conditions, as well as challenges toward reproducing long-term slowly progressive human disorders under conditions of short-lived experimental animals. This paradox in clinical trials, where muscle mass increases without proportional functional gains, highlights a fundamental limitation in our understanding of muscle quality versus quantity. Myostatin inhibitors exemplify this issue: despite consistently inducing hypertrophy, they often fail to produce equivalent improvements in strength or physical capacity. This discrepancy is thought to arise from compensatory activation of other TGF-β superfamily members, impaired tendon–muscle coupling, and the gap between muscle hypertrophy and neuromuscular performance. Similarly, high-profile phase II/III failures of selective androgen receptor modulators (SARMs) and myostatin inhibitors underscore the translational hurdle, as preclinical success rarely translates into clinically meaningful functional outcomes. Patient heterogeneity represents another major barrier to therapeutic translation. Variability in age, comorbidities, genetic background, and metabolic profile produces markedly different responses to identical interventions (Liu et al., 2024). Such heterogeneity complicates clinical trial outcomes and highlights the importance of developing stratified and personalized therapeutic approaches guided by molecular and functional biomarkers. Genetic difference of muscle metabolism, inflammatory response, as well as drug metabolism, generate varied treatment response, however, treatment regimens currently available seldom take these distinctions into consideration (Zhang et al., 2021). Pattern ofcomor-bity has immense influence upon muscle wasting progression as well as response to treatment, as well as diseases such as diabetes, cardio-vascular diseases, as well as chronic kidney conditions, generate sophisticated metabolic milieu, capable of triggering or suppressing treatment efficacy (Boccardi, 2024). Multiplicity ofchronic conditions also renders treatment choosing complicated due to drug interactions, contraindications, as well as alternative treatment needs.

### 6.2 Research priorities and emerging technologies

To advance muscle wasting therapeutics, there is an imperative for strategic research prioritization that fills existing gaps of understanding but also takes advantage of emerging technology (Liu et al., 2024). Biomarker discovery remains an area of continued highest priority, where markers that not only diagnose but also predict treatment response, follow progression of the disease, and identify pre-destinated individuals for dramatic muscle loss are needed (Hsu et al., 2024; Qazi et al., 2019). Current research generates multi-marker panels of inflammatory cytokines, metabolic markers, microRNAs, and lipidomic signatures to develop broad molecular profiles. Interleaving artificial intelligence and machine algorithms for interpretation of these complex sets of variables has the promise of creating new biomarker sets and predicting individual trajectories of the disease with unprecedented accuracy. Combinatorial treatment regimens represent a promising research direction, particularly given the limitations of single-target approaches (Lee et al., 2024). However, translational challenges persist, as simplified and short-lived animal models insufficiently replicate the multifactorial, chronic progression of human muscle wasting disorders. Moreover, most preclinical studies focus on muscle hypertrophy, whereas clinical trials demand functional endpoints such as strength, endurance, and quality of life. Designing trials that bridge this gap remains a key challenge. Systematic characterization of synergistic pairs is an innovation that needs new trial designs and extensive outcomes measures. Digital health technologies have research transformation possibilities, as they do for practice, because physical activity, activation of muscles, and metabolic measures are continuously being measured and tracked by wearables that give us never-before-seen insights into out-of-center, true-world functional capacity (Yang et al., 2024). Adoption of these technologies into trials could allow for remote monitoring, decreased participant burden, and endpoints that are more ecologically valid. Prevention strategies for muscle wasting have received insufficient attention despite the potential for greater impact than treating established disease, with research priorities including identifying modifiable risk factors across the lifespan and developing risk prediction models. Single-cell and spatial transcriptomics technologies are revealing unprecedented heterogeneity within muscle tissue, identifying distinct cell populations and their interactions in health and disease (Lai et al., 2024). These insights challenge traditional views of muscle as a homogeneous tissue and open opportunities for targeting specific cell types or intercellular communication pathways. The integration of multi-omics data at single-cell resolution will provide systems-level understanding of muscle wasting mechanisms and identify novel therapeutic targets (Yang et al., 2024). Advances in tissue engineering and regenerative medicine promise to move beyond symptomatic treatment toward true muscle regeneration, with three-dimensional bioprinting, biomimetic scaffolds, and *in vivo* reprogramming approaches representing frontier technologies (Chung et al., 2025; Cai et al., 2022). The emergence of senotherapeutics targeting cellular senescence opens new avenues for addressing age-related muscle wasting, with early clinical trials showing promise though muscle-specific effects require further investigation. The management of muscle wasting is complicated by its heterogeneous etiologies, including age-related sarcopenia, cachexia in chronic disease, immobilization-induced disuse atrophy, and nutrition-related muscle loss. As each form arises from distinct, although overlapping, pathophysiological processes, “one-size-fits-all” interventions often result in modest or inconsistent outcomes. Recent advances in multi-omics technologies offer unprecedented potential to refine diagnosis and stratify patients. Integrated analyses of genomics, transcriptomics, proteomics, metabolomics, and lipidomics can contextualize molecular signatures characteristic of distinct subtypes. For instance, metabolomics has highlighted sarcosine deficits in sarcopenia that link macrophage polarization to impaired regeneration, while lipidomic profiling identifies ceramide accumulation in obesity-driven sarcopenia (Liu et al., 2025). Incorporating such molecular “fingerprints” into diagnostic workflows could permit early-stage identification of individuals at risk, and inform the choice of nutritional, pharmacological, or regenerative interventions.

### 6.3 Future perspectives and implementation strategies

The complexity of muscle wasting demands evolution from fragmented, specialty-specific care toward integrated models that address the multifaceted needs of affected individuals (Liu et al., 2024). Future care models must seamlessly integrate primary care screening, specialist evaluation, rehabilitation services, nutritional support, and psychosocial care within coordinated frameworks. The development of multidisciplinary muscle health clinics could provide comprehensive assessment and treatment while serving as hubs for clinical research and education. Healthcare system integration requires fundamental changes in how muscle wasting is conceptualized and prioritized within medical practice (Sayer et al., 2024). Electronic health record integration of validated screening tools, automated risk alerts, and clinical decision support systems could facilitate systematic identification and management of at-risk patients. The economic sustainability of muscle wasting interventions necessitates demonstration of value through reduced healthcare utilization, prevention of disability, and maintenance of independence (Liu et al., 2024). Health economic modeling suggests that even modestly effective interventions could provide substantial cost savings through reduced hospitalizations, nursing home admissions, and caregiver burden. However, current reimbursement models often fail to incentivize preventive care or multidisciplinary interventions, requiring policy reforms that align payment structures with long-term outcomes. Patient empowerment and involvement are key features of innovative models of care, as programs of training encourage intervention that has been implemented, modifiable risk factors, and muscle health (Shen et al., 2023). Health websites that have customized progress tracking, nutritional counseling, and physical exercise programs can have care removed from the conventional office settings. Universality of muscle wasting and globality of population ageing justify cross-national working and sharing of findings of research (Ebner et al., 2020). Standardisation of outcome measures, of diagnosis, and of composition of international research groups can quicken pace whilst allowing results to have generalizability across varied populations. Technology transfers and capacity-building measures are required for ensuring that benefits accrue to those middle- and low-income countries where rapid expansion of the burden of muscle wasting is creating an imperative for effective intervention. Success in management of these challenges will entail sustained leadership by those who practise clinically, those who have responsibility for health policy, those who have responsibility for research, and by society. Through collaborative efforts yielding to the varied biological, clinical, and social characteristics of muscle wasting, the coming decade can have hope for re-directing this area of research from one of limited options and untoward outcomes to one of hope and productive intervention for all those affected.

Several systemic barriers remain in advancing muscle wasting research and therapy. Cross-study variability in definitions, endpoints, and outcome measures hampers synthesis of evidence. Patient heterogeneity—including genetic background, comorbidities, and lifestyle—complicates trial design and interpretation, limiting the generalizability of findings. There is also a lack of longitudinal studies assessing quality of life and functional endpoints beyond muscle mass. Furthermore, the economic and ethical challenges surrounding advanced therapies (cellular, gene-based) prevent their equitable implementation. Going forward, greater emphasis on standardized definitions, global collaborative research networks, and integration of digital health platforms could provide the foundation for achieving meaningful clinical progress.

## 7 Conclusion

Muscle wasting is an extremely complex, multifactorial condition of enormous significance for mankind’s health and healthcare delivery around the globe. This broad-scoping analysis has integrated existing framings of the pathophysiological processes of the various forms of muscle wasting, i.e., sarcopenia, cachexia, disuseatrophy, and neuromusculardisease. Protein anabolism and catabolism, inflammatory signaling, mitochondrial perturbations, hormonal perturbations, and neural influences each interact complexly to produce an unforgiving therapeutic environment that demands multitargeted strategies. Even as there have been enormous increases by extending methodologies of diagnosis, from tests of function to promising biomarkers and advanced imaging, and an expanded armamentarium of treatment including nutritional therapy, innovative drugs, exercise rehabilitation, and hopeful regenerative medicine strategies, there are enormous gaps between advances and translation into conventional use.
